# Cookery and Health

**Published:** 1861-12

**Authors:** 


**COOKERY AND HEALTH.**—Next in importance to having something to eat, is the proper preparation of it for the table. Bad cookery kills multitudes outright, and robs other multitudes of half the pleasure of life. Soyer was considered the most accomplished cook that ever lived in London; his experiences are embodied in the twenty pages, which are a part of our little volume on "Soldier Health," sent for twenty-five cents, a book which we think every person who has a relative or near friend in the army ought to send to the same, even if its price had to be procured by the loss of a dinner. Of this book an unknown correspondent writes: "A young brother has just gone into camp; but before he started, a God-send came—your book on 'Soldier-Health.' My father has presented one to each mess of a company, and sent one to the Colonel, recommending its introduction through the regiment." A sick soldier is worse than nobody, for he throws a burden on a well one; hence, to keep him well is a matter of prime consideration.

